# COVID-19 Vaccines: A Review of the Safety and Efficacy of Current Clinical Trials

**DOI:** 10.3390/ph14050406

**Published:** 2021-04-25

**Authors:** Zhi-Peng Yan, Ming Yang, Ching-Lung Lai

**Affiliations:** 1 Department of Medicine, Queen Mary Hospital, The University of Hong Kong, Hong Kong 999077, China; 2 Department of Ophthalmology, The University of Hong Kong, Hong Kong 999077, China; hrmeym@hku.hk

**Keywords:** COVID-19, vaccine, safety, efficacy, herd immunity

## Abstract

Various strategies have been designed to contain the COVID-19 pandemic. Among them, vaccine development is high on the agenda in spite of the unknown duration of the protection time. Various vaccines have been under clinical trials with promising results in different countries. The protective efficacy and the short-term and long-term side effects of the vaccines are of major concern. Therefore, comparing the protective efficacy and risks of vaccination is essential for the global control of COVID-19 through herd immunity. This study reviews the most recent data of 12 vaccines to evaluate their efficacy, safety profile and usage in various populations.

## 1. Introduction

The COVID-19 pandemic is caused by severe acute respiratory syndrome coronavirus 2 (SARS-CoV-2). Up till February 2021 it had infected more than 110 million patients, causing 2.4 million deaths worldwide, according to data recorded by the World Health Organization (WHO) [1].

The prevention and control of the epidemic in 2020, other than treatment of symptomatic patients, has included monitoring of asymptomatic infections, follow-up and monitoring after cure and discharge, close contact tracking, high-risk population screening, and disinfection of the epidemic source, but the only way for the radical control of COVID-19 infections is by effective vaccination. Vaccines stimulate the body to produce specific antibodies, with anamnestic response when the body is exposed to this pathogen again.

During 2020, there has been extensive research to look into the use of vaccinations to prevent further transmission of SARS-CoV-2. Globally, several prospective vaccines have been produced and used by the public (Table 1). The protective efficacy and immunogenicity profile of each vaccine is also documented (Table 2).

There are currently two forms of messenger ribonucleic acid (mRNA) vaccines: non-replicating mRNA (NRM) vaccines and self-amplifying mRNA (SAM) vaccines. The constructed mRNA is formulated into a carrier—usually lipid nanoparticles—to protect them from degradation and promote cellular uptake [2]. After the carrier particles are ingested into the cell, mRNA is released, which is translated by the ribosome to produce the target protein (recognizable antigen) [3]. After the target protein is secreted by the cell, it is rec-ognized by the immune system and stimulates an immune response.

**Figure 1:**
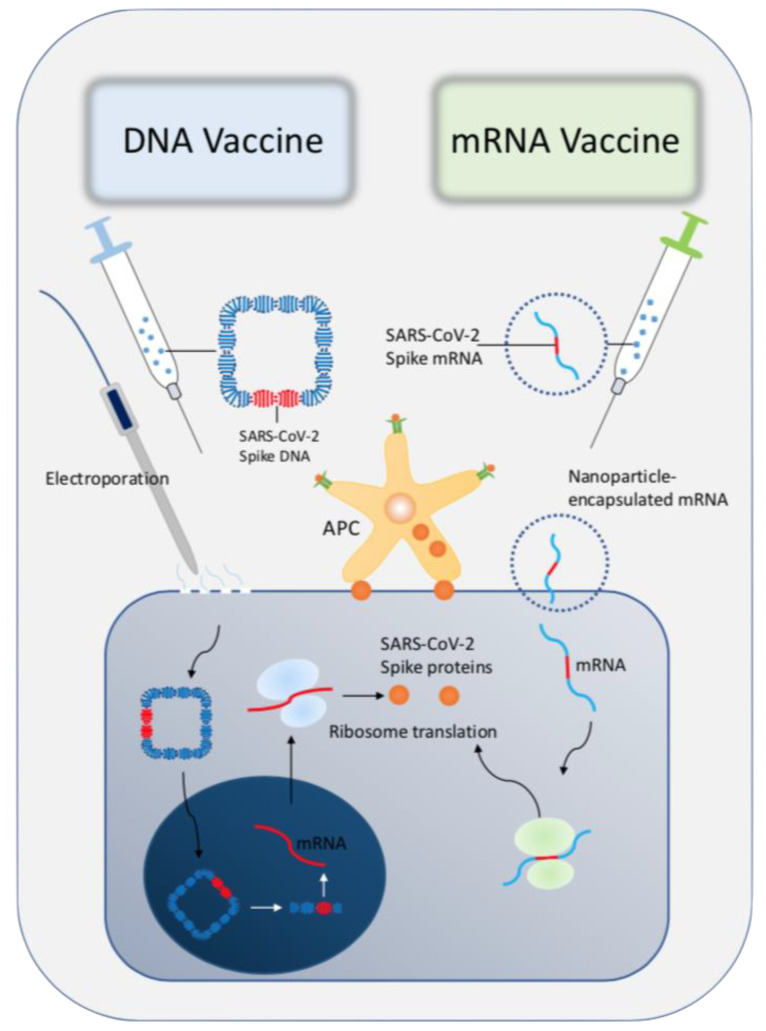
Schematic graph of the comparison between DNA and mRNA vaccine in terms of mechanisms. DNA vaccine is a circle DNA which contains the spike gene of SARS-CoV-2. After electroporation, cell membrane permeation will be increased, allowing DNA enter into cytoplasm thereby reaching to the nuclear. Subsequently, DNA will be translated into mRNA, which will be further translated into SARS-CoV-2 spike proteins and express on cell membrane. Nanoparticle-encapsulated mRNA encoding SARS-CoV-2 antigen will be integrated into cytoplasm. The spike mRNA utilizes ribosome and bases to translate spike proteins, which express on the cell membrane. The membrane spike protein will be recognized by antigen presenting cell (APC) thereby activating immune reaction.

DNA vaccines, also known as nucleic acid vaccines or genetic vaccines, have also been studied. DNA vaccines are eukaryotic expression plasmid DNA (sometimes also RNA) that encode immunogens or immu-nogens4. It can enter animals through a certain route, and be transcribed and translated after being taken up by host cells. The antigen protein can stimulate the body to produce two kinds of non-specific and specific immune responses, thereby playing a role in immune protection [33,34]. The production process of mRNA is not complicated. The difficulty lies in the fact that mRNA is prone to folding and failure in the absence of protection [35]. Therefore, there is the shortcoming of extremely poor stability. It is questionable whether unstable mRNA is safe for the human body [36]. The comparison between DNA and RNA vaccines is shown in Figure 1.

As of 10 April 2021, the top five countries with vaccination programs are the United States (6.129 million), China (4.052 million), the European Union (2.66 million), the United Kingdom (1.82 million) and India (1.084 million) [37]. Although the implementation of vaccination is one of the important factors to achieve global herd immunity, there is no consensus concerning the superiority of one vaccine over the others in terms of protective efficacy and safety profile, even thigh previous reviews have commented on some of the vaccines [38,39].

To date, there are 86 vaccines under development in clinical phase trials. They are developed with different methods such as protein subunits, inactivated virus, DNA-based vaccine, RNA-based vaccine, viral vectors, and live-attenuated viruses. (see Table 3) [40]. However, many of them are currently in preclinical or phase 1 trials, or without publishing on academic journals at the time of writing. The inclusion criteria of this review are: (1) vaccines that has at least finished their phase 2 clinical trials; and (2) the clinical data of the trial has been published in academic journals and accessible on databases (PubMed, Embase, MedLine, Cochrane) at the time of writing. Exclusion criteria includes: (1) vaccines that are on preclinical phases at the time of writing. (2) vaccines that have not gone through at least phase 2 trials 3) vaccines that have phase 2 trials but have not published their data in academic journals nor accessible on databases (PubMed, Embase, Medline, Cochrane).

This study reviews 12 vaccines in production to evaluate their protective efficacy, safety profile and usage in high risk populations such as children, elderly and patients with co-morbidities.

## 2. BiONTech (BNT162b1 and BNT162b2)

The BiONTech trials focus on two candidates: BNT162b1 and BNT162b2. Both vaccines are lipid-based, nucleoside-modified mRNA vaccines that encode the trimerized receptor-binder (RBD) of the spike glycoprotein SARS-CoV-2. The RBD-IgG concentrations and SARS-CoV-2 neutralizing titres were measured after complete course of the vaccines. In the trial of BNT162b112, serum IgG geometric mean concentra-tion (GMC) of the recipient after first dose was comparable to the convalescent sera of COVID-19 patient. The trial showed a strong, dose-dependent vaccine-induced antibody response: the GMC of vaccine recipients is 8 times and 42 times the convalescent sera in the 10 μg and 30 μg group, respectively. A further increase to 100 μg showed no additional elevation of RBD IgG concentration, compared with 10 μg and 30 μg trials [4,5]. 

BNT162b1 induced functional CD4^+^ and CD8^+^ T cell responses in almost all recipients: 95.2% participants mounted RBD-specific CD4^+^ T cell responses. There is a positive correlation between RBD-binding IgG and SARS-CoV-2 neutralizing antibody titres [6]. Severe adverse events, such as grade 3 decrease of lymphocyte count and grade 2 neutropenia, were manageable. No clinical deteriorations were observed.

The overall serological responses of BNT162b2 and BNT162b1 were similar [7]: Phase 2/3 trial showed they conferred 94.6% (95% CI 89.7–97.3) protection against COVID-19 in persons older than 16 years of age [8]. Double dose vaccination further boosts the immune response in both younger and older adults, while the response was weaker in participants 65 to 85 years old. Exploration of dose elevations of vaccinations in the elderly should be conducted in future research.

Serious adverse events such as death from arteriosclerosis and cardiac arrest, paroxysmal ventricular arrhythmia were recorded. However, cardiovascular events occurred similarly in the placebo group, with two deaths due to haemorrhagic stroke and myocardial infarction, and two with unknown causes. It is uncertain whether the vaccine increases cardiovascular risk.

COVID-19 infections is associated with a higher inflammatory burden that can induce vascular inflammation, myocarditis and cardiac arrhythmias [17]. Vaccinations for other acute respiratory virus infection show the possibility of a transient increase in the risk of vascular events [18]. Some studies showed a 10-fold increase of acute myocardial infarction admission within the seven days for of testing positive for influenza B, and a 5-fold increase of risk with influenza A [41,42,43]. Another study suggests that binding of SARS-CoV-2 to ACE2 can cause acute myocardial and lung injury through the alteration in ACE2 signaling pathways [44]. The effect of vaccinations for patients with pre-existing cardiovascular diseases have to be further elucidated.

## 3. Moderna (mRNA1273)

mRNA1273 is manufactured by Moderna. It encodes stabilized prefusion S-2P antigen, consisting of the SARS-CoV-2 glycoprotein with a transmembrane anchor and an intact S1-S2 cleavage site [9]. A preliminary report showed the binding antibody IgG GMT to S-2P increased after vaccinations, with 100% serocon-version rates by day 15. Dose-response relationship was observed with higher dosage eliciting stronger IgG GMT. Both low dose (25 μg) and medium dose (100 μg) elicited CD4^+^ T cell responses by expression of Th1 cytokines.

The phase 1 clinical trial showed a dose-response relationship [45]. It also elicited a strong CD4^+^ cytokine response involving Th1 helper T cells. The higher dosage (100 μg) was chosen for phase 3 clinical trials. Robust neutralizing activity to the 614G variant was observed for the 100 μg dose, regardless of the patients’ age.

The phase 3 clinical trial showed 94.1% (95% CI 89.3–96.8; *p* < 0.001) protective efficacy in preventing COVID-19 illness [10]. The vaccine efficacy to prevent COVID-19 was consistent across subgroups stratified by age (18 to <65 years of age and ≥65 years), presence of risk for severe COVID-19, sex, and race and ethnic groups. The frequency of grade 3 adverse events in the placebo group (1.3%) was similar to that in the vaccine group (1.5%).

## 4. ChadOx1 nCoV-19 (AZD1222)

ChadOx1 nCoV-19 consists of replication-deficient simian adenovirus vector ChAdOx1, containing the full-length structural surface glycoprotein of SARS-CoV-2, with a tissue plasminogen activator leader sequence [12]. It expresses a codon-optimised coding sequence for the spike protein. Upon vaccination, antibodies against SARS-CoV-2 spike protein peaked by day 28 and remained elevated up to day 56 in participants receiving 1 dose. The median titre of the booster-dose group was more than five times higher than the single-dose group. Paracetamol was used to reduce local regional side effects such as fever and myalgia. Prophylactic paracetamol was prescribed in certain participants, but serological response was independent of prophylactic paracetamol prescription.

ChAdOx1 nCoV-19 appears to be better tolerated in older adults than in younger adults, and it provides similar immunogenicity across all age groups after a booster dose [13]. Serological response was independent of dosage and age after booster, with the IgG level being consistently higher than those without booster vaccinations. Median IgG titres peaked by day 42 in most groups who received two-dose vaccinations. A higher vaccine efficacy was observed when the participants first received a low-dose followed by a stand-ard-dose (90%, 95% CI 67.4–97.0, *p* = 0.01), compared with two standard-dose recipients (62.1%, 95% CI 41.0–75.7) [24].

In terms of safety profile, 13 serious adverse events occurred but none was considered related to either study vaccine as assessed by the investigators [13]. There was a reported case of hemolytic anemia and three cases of transverse myelitis. The independent neurological committee considered two of them were unlikely to be related to vaccination, and one of them was an idiopathic, short segment spinal cord demyelination [14].

Phase 3 trials are being performed in the United Kingdom, Brazil and the United States of America to assess the protective efficacy and safety [13].

Various thromboembolic events were reported after participants have received ChadOx1 nCoV-19 (AZD122) vaccinations. One of the reasons may be related to post-vaccination immune-mediated thrombo-cytopenia [46]. In a report including 28 patients after receiving AZD122 with thromboembolic events, all of them were tested positive for anti-platelet factor 4(PF4)-heparin antibodies, which clinically mimics auto-immune heparin-induced thrombocytopenia [47]. This was similarly observed in another study where five participants with thromboembolic events (100%) tested positive with high level of IgG anti-PF4-polyanion complexes, measured by enzyme linked immunoassay (ELISA) [48]. The adverse reaction may be related to the adenovirus-platelet-leukocyte complexes formed after vaccination, which are taken up by the liver by interaction [28] with membrane-associated heparan sulphate proteoglycan (MAHSP) [49,50]. MAHSP acts as a receptor for viral entry. Heparin can lead to dose-dependent inhibition of this reaction, leading to induction of anti-PF4/heparin antibodies [51]. Subsequently, heparin-induced thrombocytopenia and thrombophilia was observed in patients after receiving AZD122 vaccination.

## 5. Convidecia (Adenovirus Type-5 Vectored COVID-19 Vaccine)

Adenovirus type-5 (AD-5) vectored COVID-19 vaccine is a replication of defective Ad5-vectored vaccine expressing the spike glycoprotein SARS-CoV-2 [15]. It clones an optimized full-length spike gene based on Wuhan-Hu-1 with the tissue plasminogen activator signal peptide gene into an E1 and E3 deleted Ad-5 vector, and constructed the Ad-5 vectored COVID-19 vaccines using the Admax system. The vaccine demonstrated a dose-response relationship at day 28 after vaccination: the T-cell responses in the high dose group were significantly higher than that in the low-dose group (*p* < 0.0010), but not significant compared with that in the middle group. TNF-α expression from CD4^+^ T cells was significantly lower in the low dose group than in the high dose (*p* < 0.0001) and middle dose groups (*p* = 0.0032). TNF-α expression from CD8^+^ T cells was higher in the high-dose group than that in both the middle dose group (*p* = 0.016) and the low-dose group (*p* < 0.0001).

The phase two trial showed a higher dosage correlates with a higher seroconversion rate and higher GMTs of neutralizing antibody responses to pseudovirus [16]. The seroconversion rate in high-dose group was 59% (95% CI 52–65) and 47% (95% CI 39–56). The GMT were 61.4 (95% CI 53.0–71.0) in the high-dose group and 55.3 (95% CI 45.3–67.5) in the low dose group. Stratified analysis based on age showed older adults (>55 years) were associated with lower antibody responses in both dose groups post-vaccinations. A total of 25 grade 3 or above adverse events were documented, but they were self-limiting and resolved within 3 to 4 days without medications.

Phase 3 trial are being performed globally, with 40,000 participants. It is expected to be completed by January 2022 [17].

## 6. Gam-COVID-Vac (Recombinant Adenovirus Type 26 and Recombinant Adenovirus Type 5 Vaccine)

rAd26-S and rAD5-S are vaccines made by Russian manufacturer which carry the gene for SARS-CoV-2 full-length glycoprotein S. Phase 1/2 studies showed both rAd26-S and rAD5-S formulations were safe and well tolerated [18]. Patients receiving combined rAD26-S and rAD5-S were associated with a higher se-roconversion rate (100%) and neutralising antibody GMT (49.25) on day 28 [19]. Combined regimen was better than individual rAD26-S or rAD5-S injection. Increased CD4^+^ T cells, CD8^+^ T cells and IFN- γ secre-tion were observed in all vaccine recipients. No serious adverse events were reported.

The phase 3 study showed a protective efficacy of 91.6% (95% CI 85.6–95.2) against COVID-19 [19]. Immunogenicity was significantly higher in the vaccination arm: The RBD-specific IgG was detected in 98% participant samples, with a GMT of 8996 (95% CI 7610–10,635) and a seroconversion rate of 98.25%. Conversely, the RBD-specific IgG was detected in 15% participant samples with a GMT of 30.55 (95% CI 20.18–46.26) and a seroconversion rate of 14.91% (*p* < 0.0001 vs. the vaccination arm). Neutralising antibody follows a similar trend too: with GMT of 44.5 (95% CI 31.8–62.2) and seroconversion rate of 95.83% in the vaccination arm; compared with GMT of 1.6 (95% CI 1.12–2.19) and 7.14% seroconversion rate.

## 7. Covovax (NVAX-CoV2373)

NVAX-CoV2373 is a recombinant SARS-CoV-2 nanoparticle vaccine composed of trimeric full-length sARS-CoV-2 spike glycoproteins and Matrix-M1 adjuvant. The phase 1 study showed two-dose 5 μg regimen with adjuvant induced IgG GMT and neutralization responses that exceeded convalescent serum from most symptomatic COVID-19 patients [20]. The immunological outcomes in 5 μg and 25 μg vaccination groups were comparable. Second vaccinations with adjuvant resulted in GMT level four times greater than the convalescent plasma in symptomatic patients. Adjuvant regimens induced polyfunctional CD4^+^ T-cell responses that were reflected in IFN-γ, TNF-α and IL-2 production on spike protein stimulation. No serious adverse events were reported. Interim analysis showed the vaccine achieved protective efficacy of 86% against UK variant and 60% against South Africa variant [21]. The phase 3 trial showed a protective efficacy of 89.3% (95% CI 75.2–95.4) against B.1.1.7 UK variant, but only 49.4% (95% CI 6.1–72.8) against B.1.351 variant [22].

## 8. WIV04-Strain Inactivated SARS-CoV-2 Vaccine

The WIV-04 strain inactivated SARS-CoV-2 vaccine is designed by the Wuhan Institute of Biological Products Co Ltd. The WIV-04 strain was isolated and cultivated in a Verco cell line for propagation, and the supernatant of the infected cells was inactivated by β-propiolactone. Interim analysis of two randomised controlled trials showed a seroconversion rate of 100% in phase 1 trial and 85.7% in the phase 2 trial [10]. A lower-dosage injection was associated with a higher GMT of neutralizing antibody at day 14 after the third injection, compared with other dosage groups. Injection schedule on day 0 and 21 confer a higher GMT, compared with the schedule of day 0 and 14. Most patients started to generate antibody response after the second injection, and remained at high level 14 days after the third injection. The most common adverse reactions were injection site pain and fever, which were mild and self-limiting. The phase 3 study data was not available at the time of writing.

## 9. BBIBP-CorV

BBIBP-CorV is developed by the Beijing Institute of Biological Products. It is an inactivated vaccine developed from the strain 19nCoV-CDC-Tan-HB02 (HB02) [11]. The HB02 strain was purified and passaged in Vero cell lines to generate vaccine production by using a novel carrier in a basket reactor. In the phase 1 trial, a higher dosage (8 μg) was associated with a higher seroconversion rate by day 14, while seroconversion rates reached 100% in all three dosage cohorts on day 28. By day 28, the neutralizing antibody GMT was significantly higher in the high-dose group than the low-dose group (2 μg), with no significant difference between medium-dose (4 μg) and high-dose. Younger adults were associated with higher neutralizing anti-body GMT, compared with older adults (>60 years).

The phase 2 trial showed the immunization schedule of 4 μg on day 0 and 21 was associated with the highest neutralizing antibody GMT (282.7, 95% CI 221.2–361.4), compared with other immunization schedules. One grade 3 or above adverse event was documented due to self-limiting grade 3 fever (>38.5 °C).

A phase 3 study is currently underway in Abu Dhabi with 15,000 participants: 5000 participants receiving placebo, another 5000 receiving BBIBP-CorV, and the remaining 5000 receiving another inactivated vaccine manufacturer by Sinopharm [23].

## 10. Coronavac Vaccine

Coronavac is developed by Sinovac Life Sciences (Beijing China) as an inactivated vaccine created from Vero cells that have been inoculated with SARS-CoV-2 (CN02 strain) [24]. The phase 1 trial showed seroconversion rates of 88% and 100% and 8% in the 3 μg, 6 μg and placebo groups on day 28, respectively. The neutralising antibody GMT were 465.8 (95% CI 288.1–753.1), 1395.9 (95% CI, 955.2–2039.7) and 89.8 (95%CI 76.1–105.9) in the three groups, respectively. Higher dosage was associated with a better immunogenicity.

The phase 2 immunization schedule trial showed receiving vaccination on day 0 and 14 resulted in the most promising outcomes: seroconversion rates were 97%, 100% and 0% in the 3 μg, 6 μg and placebo groups on day 28, respectively. The neutralising antibody GMT were 44.1 (95% CI 37.2–52.2), 65.4 (95% CI 56.4–75.9) and 2.0 (95% CI 2.0–2.1) in the three groups, respectively. One case of serious adverse events related to acute hypersensitivity with presentation of urticaria 48 h after the first dose. It was managed with chlorphenamine and dexamethasone, and recovered within 3 days.

The phase 3 study data has not been published in medical journals. An online search of the phase 3 study in Brazil showed a 50.4% protective efficacy in preventing symptomatic infections, 78% protective efficacy in preventing mild cases requiring treatment and 100% prevention of severe cases [52]. Phase 3 studies in Turkey and Indonesia showed a protective efficacy of 83.5% and 65.3%, respectively [53,54].

## 11. Ad26.COV2.S

Ad26.COV2.S is developed by Johnson & Johnson. It is a recombinant, replication-incompetent adenovirus serotype 26 (Ad26) vector encoding a full-length and stabilized SARS-CoV-2 spike protein. Early animal studies showed promising efficacy with low-dose single-shot vaccination [25,26]. In the phase 1 clinical trial, binding and neutralizing antibodies were detected in 100% of vaccine recipients by 57 days after single vaccinations [27]. The geometric mean titres (GMT) of spike-specific binding antibodies and neutralizing antibodies ranged from 2432–5729 and 242–449, respectively. A booster immunization on day 57 increased binding antibody titres and neutralizing antibody titres by a mean of 2.56-fold (range 1.58–3.04) and 4.62-fold (range: 3.56–5.68), respectively. An interim study showed the titres remain stable until at least day 71 [28]. Strong immune responses were recorded as CD4^+^ T cells were detected in 76 to 83% of the young patients (aged 18–55 years), and 60 to 67% in older patients (aged greater than 65). Phase 3 data showed a 66.9% (95% CI 59.0–73.4) protective efficacy across all participant age groups, and 76.3% (95% CI, 61.6–86.0) in participants older than 60 years old [29]. In preventing severe or critical COVID-19, Ad26.COV2.S was associated with 76.7% efficacy at 14 days, and 85.4% at 28 days. Adverse reactions were recorded such as thromboembolic events (15 in vaccination arm and 10 in placebo arm) and tinnitus (6 vs. 0).

Subgroup analysis based on region showed a higher vaccine efficacy in N. America, compared with South Africa and Latin America. The protective efficacies were 74.4% (95% CI 65.0–81.6) at 14 days and 72.0% (95% CI 58.2–81.7) at 28 days; compared with 52.0% (95% CI 30.3–67.4) at day 14 and 64% (95% CI 41.2–87.7) in South Africa. The protective efficacies in Latin America were 64.7% (95% CI 54.1–73.0) and 61.0% (95% CI 46.9–71.8), respectively. This may be related to the difference in the prevalence of mutant strain of SARS-CoV-2 in different regions.

## 12. Covaxin (BBV 152)

BBV 152 is a whole-viron inactivated SARS-CoV-2 vaccine formulated with a toll-like receptor 7/8 agonist molecule (IMDG) adsorbed to alum (Algel) [30]. It is developed by Bharat Biotech from an isolated NIV-2020-770 strain of a patient with COVID-19 sequenced in India. Previous animal studies showed acceptable safety profiles, humoral and cell-mediated responses [31]. Phase 2 trials showed a good reactogenicity, safety profile, and enhanced humoral and cell-mediated immune responses when participants received a higher dose (6 μg) of Algel-IMDG formulation [32]. In the phase 2 trial, the GMT at day 56 was significantly higher in the 6 μg group (197.0, 95% CI 155.6–249.4) compared with the 3 μg group (100.9, 95% CI 74.7–137.4, *p* = 0.0041). Seroconversion rates were 92.9% (95% CI 88.2–96.2) in the 3 μg group, and 98.3% (95% CI 95.1–99.6) in the 6 μg group. The Algel-IMDG formulation elicited T-cell responses biased to a Th1 phenotype at day 42, with no significant difference in causing local or systemic adverse reactions between the 3 μg and the 6 μg groups. No serious adverse events were reported in the study. Protective efficacy was not reported.

## 13. Challenges

In view of the surging infections and promising efficacy in clinical trials of vaccines (Table 2), many countries have advocated vaccination programs for their citizens. However, questions have been raised concerning the efficacy against new variant strains. Experience in Manaus (Brazil) showed secondary immunity alone was not sufficient to arrest transmission [55], possibly due to new variant strains. The B.1.1.7 of the UK and South African 501Y.V2 variants are shown to cause alterations to the spike protein, which may affect immune recognition of antibodies derived from existing vaccines [56]. Further clinical trials are required to test for the efficacy of existing vaccines against mutant variants.

Another problem is the duration of the protective efficacy. It is likely that at least yearly boosters are necessary. Seasonal modification to annual vaccines to arrest the transmission of previous strains may also be considered. It is also doubtful whether circulating neutralizing antibody is protective against COVID-19 infection as animal studies showed robust viral infective activities in nasal turbinate. Reinfection is still potentially possible [57].

Also with the expansion of the vaccination programs in the general population, the relationship of certain side effects, such as the thrombotic events occurring after receiving ChadOx1 nCoV-19, with the vaccines has to be further determined.

The pathological correlation between incidence of cardiovascular adverse events and vaccination with in-activated or live-attenuated virus has to be elucidated. SARS-CoV-2 infection is associated with systemic inflammatory response causing cytokine releases and cytokine storm, resulting in vasculopathy and its complications [58]. Likewise, influenzae carries similar pathogenesis as SARS-CoV-2. However, the experience of influenzae vaccinations (inactivated virus) shows that vaccinations reduced major cardiovascular events significantly, and has become part of the routine care of patients with chronic cardiovascular conditions [59]. COVID-19 vaccinations do not follow the typical trend of influenzae. In general, attenuated patho-gens have the very rare potential to revert to its pathogenic form [60]. Further studies is required to determine whether vaccines with inactivated SARS-CoV-2 can reduce or induce cardiovascular events.

Diabetic patients are associated with a higher risk of inflammatory response and coagulopathy during an infection episode [61]. Close monitoring of inflammatory markers, tight glycemic controls and lifestyle modifications are recommended for diabetic COVID-19 care [62]. Acute complications after vaccinations can be monitored by measurement of prognostic inflammatory markers, such as serum ferritin, lactate dehydrogenase, C-reactive protein (CRP), erythrocyte sedimentation rate, D-dimer level, cardiac troponin and N-terminal pro-brain-type natriuretic peptide (NT-proBNP) [63,64,65,66]. These markers have close associations with the prognosis of COVID-19 infections. However, the interval and duration of monitoring has to be further studied. The relation between thrombotic events and vaccine using as adenovirus vector has been discussed in a previous section.

## 14. Conclusions

The COVID-19 vaccines in clinical trials have all shown promising immunogenicity with varying degree of protective efficacy, and an acceptable safety profile. A second dose immunization gives more robust immune response in all vaccines. The immunological outcome in the elderly is poorer than in the younger recipients. Further exploration on immunization schedule is required, such as more frequent vaccinations or higher dosage in each injection. Grade 3 or above side effects are not common in the clinical trials to date.

## Figures and Tables

**Table 1:** Summary of vaccine trials.

Title [Reference]	Clinical Phase	Population Characteristics of the Latest Trial	Doses	Technology	Immunogenicity	Safety Profile
BNT162b1 [4,5,6]	1–2	45 adults in 3 groups: 10 μg, 30 μg, 100 μg 12 vaccines: 3 placebo in each group	2 injections, 21 days apart	Lipid nanoparticle nucleoside-modified mRNA vaccine, encoding the spike glycoprotein of SARS-CoV-2	Dose-dependent antibody response	No serious adverse events
BNT162b2 [7,8]	1–3	43,448 volunteers aged 16 or older in total: (1:1 ratio) 21,720 received vaccines 21,728 received placebo	2 injections of 30 μg doses for phase 3, 21 days apart	Lipid nanoparticle nucleoside-modified mRNA vaccine, encoding the spike glycoprotein of SARS-CoV-2	Similar dose-dependent response as BNT162b	No serious adverse events
mRNA-1273 [9,10,11]	1–3	30,420 adults in total: (1:1 ratio) 15,210 received vaccines 15,210 received placebo	2 injections of 100 μg doses, 28 days apart	Lipid nanoparticle capsule of four lipids, encoding the S-2P antigen.	100% seroconversion rates by day 15	Similar grade 3 adverse events in the placebo group (1.3%) and the vaccine group (1.5%)
ChAdOx1 nCoV-19 [12,13,14]	1–3	23,848 adults randomised 1:1 ratio to receive ChAdOx1 nCoV-19 or placebo	2 injections of 3.5–6.5 × 10^10^ viral particles per mL, 28 days apart	Chimpanzee adenovirus-vectored vaccine with SARS-CoV-2 spike glycoprotein	Median titre of booster-dose group is more than 5 times higher than the single-dose group.	- 13 serious adverse events - None considered related to the vaccine
Ad5-vectored COVID-19 [15,16,17]	1 & 2	508 adults randomised 2:1:1 to receive vaccine at the dosage of 1 × 10^11^, 5 × 10^10^, or placebo	1 injection	Replication defective Ad5-vectored vaccine expressing the spike glycoprotein of SARS-CoV-2	Higher antibody GMT in high-dose group, compared with medium and low-dose groups.	- 25 grade 3 or above adverse events - All resolved within 3 to 4 days without medications
rAd26-S and rAd5-S [18,19]	1–3	21,977 adults in total: 16,501 received vaccines 5476 received placebo	2 injections of 10^11^ viral particles in 0.5 mL vaccine, 21 days apart	Replication of Ad5-vectored and Ad-26 vectored vaccine expressing the gene for SARS-CoV-2 full-length glycoprotein S	100% seroconversion rate	No serious adverse events
NVX-CoV2373 [20,21,22]	1–3	30,000 adults in total: Randomised in 2:1 ratio to receive vaccine and saline placebo	2 injections of 5 mg protein with 50 mcg matrix-M adjuvant, 21 days apart.	Nanoparticle of trimeric full-length SARS-CoV-2 spike glycoproteins and Matrix-M1 adjuvant	IgG GMT and neutralization responses exceeding convalescent serum	No serious adverse events
WIV-04 strain inactivated vaccine [10]	1–2	96 adults randomised 1:1:1:1 to receive low-dose, medium-dose, high-dose and aluminium hydroxide, respectively	Phase 1: 3 injections on day 0, 28 and 56	Isolated from WIV-04 strain and cultivated in a Verco cell line, followed by serial inactivation	100% seroconversion rates in phase 1 trial and 85.7% in the phase 2	Mild injection site pain and fever (23.4%)
			Phase 2: 2 injections on day 0 and 14, or day 0 and 21			
BBIBP-CorV [11,23]	1–2	192 adults: 18–59 years (96 adults) ≥60 years (96 adults). 24 receiving vaccine of 2 μg, 4 μg or 8 μg on day 0 and 28; and 24 receiving placebo.	Phase 1: 2 injections separated 28 days	HB02-strain in Verco cell line, with serial inactivation	- Higher seroconversion with higher dosage (8 μg) by day 14, - Higher neutralizing antibody GMT in younger adults	One grade 3 adverse event: self-limiting fever (>38.5 °C)
			Phase 2: Single-dose			
Coronavac [24]	1–3	13,000 adults randomised to receive vaccine or placebo (randomisation ratio not provided)	2 injections, 28 days apart	Inactivated vaccine from Vero cell line with SARS-CoV-2 (CN02 strain)	-High seroconversion rates: 83% in the 3 μg group, 79% in the 6 μg group, and 4% in the placebo group	One case of serious hypersensitivity with urticaria, recovered 3 months after medical treatment.
Ad26.COV2.S [25,26,27,28,29]	1–3	40,000 adults randomised to receive vaccination or placebo (randomisation ratio not provided)	1 injection of 5 × 10^10^ virus particles	replication-incompetent adenovirus serotype 26 (Ad26) vector encoding full-length SARS-CoV-2 spike protein	100% seroconversion day 57	Comparable serious adverse events in vaccination group and placebo group.
BBV152 [30,31,32]	1–2	380 participants (aged 12–65 years) randomised by 1:1 ratio to receive vaccines of either 3 μg or 6 μg.	2 intramuscular injections on day 0 and day 28	whole-virion inactivated SARS-CoV-2 vaccine formulated with a toll-like receptor 7/8 agonist molecule (IMDG) adsorbed to alum (Algel)	92.9% (95% CI 88.2–96.2) seroconversion rate in the 3 μg group, and 98.3% (95% CI 95.1–99.6) in the 6 μg group.	Comparable local and systemic adverse event profile in the 3 μg (9.47%) and 6 μg (11.0%) groups. No reported serious adverse events.

**Table 2:** Efficacy and other immune responses of vaccines after completion of vaccinations.

Title [Reference]	Protective Efficacy	Antigen-Specific IgG GMT Level	Neutralizing Antibody Responses	Cellular Responses
BNT162b1 [4,5,6]	Similar to BNT162b2 (actual figure not stated)	- 10 μg: 4813 U/mL - 30 μg: 27,873 U/mL - Increase dosage to 100 μg did not increase the IgG GMC. - Lower antigen-binding IgG in participants ≥65 years of age	Higher GMT compared to convalescent serum panel - 10 μg: 1.8-fold - 30 μg: 2.8-fold	-Functional CD4^+^ and CD8^+^ responses in all participants, predominantly Th1 helper responses. - The mean fraction of RBD-specific T cells was higher than convalescent plasma.
BNT162b2 [7,8]	94.6% (95% CI 89.9–97.3)	- 10 μg: 5782 U/mL - 20 μg: 12,464 U/mL - 30 μg: 9136 U/mL - Lower antigen-binding IgG for ≥65 years of age	Higher GMT compared to convalescent serum panel 18–55 years: 1.7–4.6 times ≥65 years: 1.1–2.2 times	Not assessed
mRNA1273 [9,10,11]	94.1% (95% CI 89.3–96.8; *p* < 0.001)	- 25 μg: 299,751 U/mL - 100 μg: 782,719 U/mL - 250 μg: 1,192,154 U/mL	Neutralizing PRNT_80_ generally at or above the value of convalescent serum	- The 25 μg, 100 μg groups elicited CD4^+^ T cell responses to Th1 cytokines. - Minimal Th2 response
ChadOx1 nCoV-19 [12,13,14]	Overall: 70.4% (95% CI 54.8–80.6) 2-standard dose: 62.1% (95% CI 41.0–75.7) Low dose + standard dose: 90.0% (95% CI 67.4–97.0)	- Antigen-specific antibody peaked at day 28 with 157 GMEU - Antigen specific IgG on day 28 decreased with increasing age: 18–55 years: 6439 U/mL; 56–69 years: 4553 U/mL; and ≥70 years: 3565 U/mL	91% and 100% participants achieved PRNT_80_ responses in one-dose and booster-dose groups, respectively.	- The median SFCs PBPMC in the standard-dose groups: 18–55 years: 1187; 56–69 years: 797 ≥70 years: 977 No significant increase of PBPMC after the booster vaccination (*p* = 0.46 from paired Student’s *t* test of day 28 vs. day 42)
Ad5-vectored COVID-19 [15,16,17]	Not available at the time of writing	- High-dose: 1445.8 (95% CI 935.5–2234.5); - Medium-dose: 806 (95% CI 528.2–1229.9) - Low-dose: 615.8 (95% CI 405.4–935.5) - Seroconversions of 97%, 94% and 100% in the low-dose, medium-dose and high-dose groups, respectively.	- High-dose: 34.0 (95% CI 22.6–50.1); - Medium-dose: 16.2 (95% CI 10.4–25.2); - Low-dose: 14.5 (95% CI 9.6–12.8)) - 4-fold increase of anti-RBD IgG in 50%, 50% and 75% in the high-dose, medium-dose and low-dose groups, respectively.	- The mean SFCs PMPMC: Low-dose: 20.8 (95%CI 12.7–34.0); Medium-dose: 40.8 (95% CI 27.6–60.3) and High-dose: 58.0 (95% CI 39.1–85.9) T-cell responses in the high-dose group significantly higher than the low-dose group (*p* < 0.001)
rAd26-S and rAd5-S [18,19]	91.6% (95% CI 85.6–95.2)	SARS-CoV-2 S1 subunit-specific IgG GMT was 53,006 with Gam-COVID-Vac and 51,200 with Gam-COVID-Vac-Lyo	100% neutralizing antibody with GMT 49.25 and 45.95 by using Gam-COVID-Vac and 51,200 with Gam-COVID-Vac-Lyo, respectively.	- 100% increased formation of CD4^+^ and CD8^+^ cells, and increased IFN-γ - Median cell proliferation: In frozen formulation: CD4^+^: + 2.5% CD8^+^: +1.3% In lyophilised formulation: CD4^+^: +1.3% CD8^+^: +1.1%
NVX-CoV2373 [20,21,22]	89.3% (95% CI 75.2–95.4) against B.1.1.7 UK variant, 49.4% (95% CI 6.1–72.8) against B.1.351 South Africa variant.	- GMEU increase by 8 (15,319 units in “5 μg + M1” and 20,429 units in “25 μg + M1”). - GMEU level higher than in convalescent serum after second dose	GMFRs 5 times greater with adjuvant (5.2 times in “5 μg + M1” and 6.3 times in “25 μg + M1”). Second dose with adjuvant resulted in GMT levels 4 times greater than those in symptomatic infections.	Stimulated Th1 phenotype response with increased IFN-γ, IL-2 and TNF- α. Minimal Th2 responses as measured by IL-5 and IL-13 cytokines.
WIV-04 strain inactivated vaccine [10]	Not available at the time of writing.	- Low-dose: 415 (95% CI 288–597); - Medium-dose: 349 (95% CI 258–472); - High-dose: 311 (95% CI 229–422)	Neutralizing antibody levels increased significantly 14 days after the second dose, and the third dose	Not assessed
BBIBP-CorV [11,23]	Not available at the time of writing.	In the 4 μg trial by 14 days after the second dose, the GMTs were: - 279.2 (95% CI 192.6–404.7) against 35C; - 234.8 (95% CI 122.2–450.8) against 56Y; - 181.0 (95% CI 105.9–309.5) against 834Y; - 304.4 (95% CI 202.1–485.6) against HN97; - 117.4 (95% CI 61.1–225.4) against F13; - 193.3 (95% CI 141.4–264.0) against HB01; - 210.7 (95% CI 120.3–369.1) against BJ01, - 146.8 (95% CI 93.8–230.0) against CQ01; - 218.5 (95% CI 125.3–380.8) against QD01; - 394.8 (95% CI 256.5–607.6) against passage 7 virus.	-In age group 18–59 years, neutralizing antibody GMT were: 2 μg: 22.6 (95% CI 18.9–27.0); 4 μg: 29.3 (95% CI 23.8–36.0); 8 μg: 36.7 (95% CI 29.8–45.2) -In the age group ≥60 years, neutralizing antibody GMT were: 2 μg: 13.4 (95% CI 9.4–19.0); 4 μg: 18.9 (95% CI 13.4–26.6); 8 μg: 23.7 (95% CI 19.0–29.6)	Not assessed
Coronavac [24]	Brazil: symptomatic prevention: 50.4% - mild cases prevention: 78% Severe cases prevention: 100% Turkey: 83.5% (confidence interval not reported) Indonesia: 65.3%. (confidence interval not reported)	3 μg: 27.6 (95% CI 22.7–33.5) 6 μg: 34.5 (95% CI 28.5–41.8) Placebo: 2.3 (95% CI 2.0–2.5)	3 μg: 5.6 (95% CI 3.6–8.7); 6 μg: 7.7 (95% CI 5.2–11.5); Placebo: 2.0 (95% CI 2.0–2.0)	The average IFN-γ positive spot-forming cells per 100,000 cells were: 3 μg group: 7.4 (95% CI 3.9–11.1); 6 μg group: 3.9 (95% CI 1.0–6.7); Placebo: 1.5 (95% CI 0.2–2.9)
Ad26.COV2.S [25,26,27,28,29]	Overall: 66.9% (95% CI 59.0–73.4) ≥60 years old 76.3% (95% CI, 61.6–86.0)	- Ranged from 2432 U/mL to 5729 U/mL. - The booster immunization on day 57 increased binding antibody titres 2.56-fold (range 1.58–3.04).	- The GMT of neutralizing antibody ranged from 242 to 449. - The booster immunization on day 57 increased neutralizing antibody titres by a mean of 4.62-fold (range: 3.56–5.68).	Stronger CD4^+^ cells response recorded in younger adults: 18–55 years:76 to 83% ≥65 years: 60 to 67%
BBV152 [30,31,32]	Not reported	- 3 μg: 100.9 (95% CI 74.1–137.4) - 6 μg: 197.0 (95% CI 155.6–249.4) (*p* = 0.0041)	The neutralizing IgG GMTs at day 56 were 10,413.9 (95% CI 9142.4–11,862.2) in the 3 μg group; and 9541.6 (95% CI 8245.9–11,041.0) in the 6 μg group at day 56.	Strongly biased to a Th1 cell response at day 42. Th2 response were detected at minimal level.

GMC: Geometric Mean Concentration (U/mL); GMT: Geometric Mean Titre (U/mL); GMEU: Geometric Mean ELISA units (U/mL); GMFR: Geometric Mean Fold Rises (Times); RBD: Receptor-Binding Domain; PMPMC: Per Million Peripheral Mononuclear cells; PRNT_80_: Plaque Reduction Neutralizing Testing assay with detectable 80% live-virus neutralization.

**Table 3:** Progress of existing 86 vaccines candidates in clinical trial as at 6th April 2021.

Number	Vaccine Platform	Type of Candidate VACCINE	Usage	Developer	Clinical Status	Phase Trials Registration No.
1	Inactivated virus	CoronaVac; SARS-CoV-2 vaccine (inactivated)	2 doses (day 0 + 14) Intramuscular	Sinovac Research and Development Co., Ltd.	Phase 4	Phase ½: NCT04383574 NCT04352608 NCT04551547
						Phase 3: NCT04456595 NCT04508075 NCT04582344 NCT04617483 NCT04651790 NCT04800133
						Phase 4: NCT04756830 NCT04747821 NCT04775069 NCT04789356 NCT04754698 NCT04801888
2	Inactivated virus	Inactivated SARS-CoV-2 vaccine (Vero cell)	2 doses (day 0 + 21) Intramuscular	Sinopharm + China National Biotec Group Co + Wuhan Institute of Biological Products	Phase 3	Phase ½: ChiCTR2000031809
						Phase 3: ChiCTR2000034780 ChiCTR2000039000 NCT04510207 NCT04612972
3	Inactivated virus	Inactivated SARS-CoV-2 vaccine (Vero cell), vaccine name BBIBP-CorV	2 doses (day 0 + 21) Intramuscular	Sinopharm + China National Biotec Group Co + Beijing Institute of Biological Products	Phase 3	Phase 1/2: ChiCTR2000032459
						Phase 3: NCT04560881 NCT04510207
4	Viral vector (Non-replicating)	ChAdOx1-S—(AZD1222) (Covishield)	2 doses (day 0 + 28) Intramuscular	AstraZeneca + University of Oxford	Phase 4	Phase 1: PACTR202005681895696
						Phase 1/2: PACTR202006922165132 NCT04568031 NCT04444674 NCT04324606 NCT04684446 ISRCTN15638344 NCT04760730
						Phase 2 NCT04686773 ISRCTN69254139
						Phase 3: ISRCTN89951424 NCT04516746 NCT04540393 NCT04536051 EUCTR2020–005226-28-DE NCT04800133
						Phase 4: NCT04760132 NCT04775069
5	Viral vector (Non-replicating)	Recombinant novel coronavirus vaccine (Adenovirus type 5 vector)	1 dose Day 0 Intramuscular	CanSino Biological Inc./Beijing Institute of Biotechnology	Phase 3	Phase 1: ChiCTR2000030906 NCT04313127 NCT04568811 NCT04552366
						Phase 1/2: NCT04398147
						Phase 2: ChiCTR2000031781 NCT04566770 NCT04341389
						Phase 3: NCT04526990 NCT04540419
6	Viral vector (Non-replicating)	Gam-COVID-Vac Adeno-based (rAd26-S + rAd5-S)	2 doses (day 0 + 21) Intramuscular	Gamaleya Research Institute; Health Ministry of the Russian Federation	Phase 3	Phase 1/2: NCT04436471 NCT04437875 NCT04713488 NCT04760730
						Phase 2: NCT04587219
						Phase 2/3: NCT04640233
						Phase 3: NCT04530396 NCT04564716 NCT04642339 NCT04656613 NCT04741061
7	Viral vector (Non-replicating)	Ad26.COV2.S	1–2 doses Day 0 or Day 0+ Day 56 Intramuscular	Janssen Pharmaceutical	Phase 3	Phase 1: NCT04509947
						Phase 1/2: NCT04436276
						Phase 2: EUCTR2020-002584-63-DE NCT04535453 NCT04765384
						Phase 3: NCT04505722 NCT04614948
8	Protein subunit	SARS-CoV-2 rS/Matrix M1-Adjuvant (Full length recombinant SARS CoV-2 glycoprotein nanoparticle vaccine adjuvanted with Matrix M)	2 doses (day 0 + 21) Intramuscular	Novavax	Phase 3	Phase 1/2: NCT04368988
						Phase 2: NCT04533399
						Phase 3: NCT04611802 EUCTR2020-004123-16-GB NCT04583995
9	RNA based vaccine	mRNA -1273 mRNA-1283	2 doses (day 0 + 28) Intramuscular	Moderna + National Institute of Allergy and Infectious Diseases (NIAID)	Phase 4	Phase 1: NCT04283461 NCT04813796
						Phase 1/2: NCT04677660 NCT04712110
						Phase 2: NCT04405076 NCT04761822
						Phase 2/3: NCT04649151 NCT04796896
						Phase 3: NCT04470427 NCT04811664 NCT04805125 NCT04806113
						Phase 4: NCT04760132 NCT04792567
10	RNA based vaccine	BNT162b2	2 doses (day 0 + 21) Intramuscular	Pfizer/BioNTech + Fosun Pharma	Phase 4	Phase 1: NCT04523571 ChiCTR2000034825 NCT04816643
						Phase 1/2: NCT04588480 NCT04380701 NCT04537949 EUCTR2020-003267-26-DE
						Phase 2: NCT04649021 NCT04761822
						Phase 2/3: NCT04754594
						Phase 3: NCT04368728 NCT04713553 NCT04800133 NCT04805125 NCT04816669
						Phase 4: NCT04760132 EUCTR2021-000412-28-BE EUCTR2021-000412-28-BE NCT04780659 NCT04775069
11	Protein subunit	Recombinant SARS-CoV-2 vaccine (CHO Cell)	2–3 doses Day 0 + 28 or Day 0 + 28 + 56 Intramuscular	Anhui Zhifei Longcom Biopharmaceutical + Institute of Microbiology, Chinese Academy of Sciences	Phase 3	Phase 1: NCT04445194 ChiCTR2000035691 NCT04636333
						Phase 1/2: NCT04550351 NCT04813562
						Phase 2: NCT04466085
						Phase 3: NCT04646590
12	RNA based vaccine	CVnCoV Vaccine	2 doses Day 0 + Day 28 Intramuscular	CureVac AG	Phase 3	Phase 1: NCT04449276
						Phase 2: NCT04515147 PER-054-20
						Phase 2/3: NCT04652102
						Phase 3: NCT04674189
13	Inactivated virus	SARS-CoV-2 vaccine (Vero cells)	2 doses Day 0 + Day 28 Intramuscular	Institute of Medical Biology + Chinese Academy of Medical Sciences	Phase 3	Phase 1/2: NCT04470609 NCT04412538
						Phase 3: NCT04659239
14	Inactivated virus	QazCovid-in^®^—COVID-19 inactivated vaccine	2 doses Day 0 + Day 21 Intramuscular	Research Institute for Biological Safety Problems, Rep of Kazakhstan	Phase 3	Phase ½: NCT04530357
						Phase 3: NCT04691908
15	DNA based vaccine	INO-4800 + electroporation	2 doses Day 0 + Day 28 Intradermal	Inovio Pharmaceuticals + International Vaccine Institute + Advaccine (Suzhou) Biopharmaceutical Co., Ltd.	Phase 2/3	Phase 1: NCT04336410 ChiCTR2000038152
						Phase 1/2: NCT04447781
						Phase 2: ChiCTR2000040146
						Phase 2/3: NCT04642638
16	DNA based vaccine	AG0301-COVID19	2 doses Day 0 + Day 14 Intramuscular	AnGes + Takara Bio + Osaka University	Phase 2/3	Phase 1/2: NCT04463472 NCT04527081 jRCT2051200085
						Phase 2/3: NCT04655625
17	DNA based vaccine	nCov vaccine	3 doses Day 0 + Day 28 + Day 56 Intradermal	Zydus Cadila	Phase 3	Phase 1/2: CTRI/2020/07/026352 CTRI/2021/03/032051
						Phase 3: CTRI/2020/07/026352
18	DNA based vaccine	GX-19N	2 doses Day 0 + Day 28 Intramuscular	Genexine Consortium	Phase ½	Phase 1/2: NCT04445389 NCT04715997
19	Inactivated virus	Whole-Virion Inactivated SARS-CoV-2 Vaccine (BBV152)	2 doses Day 0 + Day 14 Intramuscular	Bharat Biotech International Limited	Phase 3	Phase 1/2: NCT04471519 CTRI/2020/07/026300 CTRI/2020/09/027674
						Phase 3: NCT04641481; CTRI/2020/11/028976
20	Protein subunit	KBP-COVID-19 (RBD-based)	2 doses Day 0 + Day 21 Intramuscular	Kentucky Bioprocessing Inc.	Phase 1/2	Phase 1/2: NCT04473690
21	Protein subunit	VAT00002: SARS-CoV-2 S protein with adjuvant	2 doses Day 0 + Day 21 Intramuscular	Sanofi Pasteur + GSK	Phase 3	Phase 1/2: NCT04537208
						Phase 2: NCT04762680
						Phase 3: PACTR202011523101903
22	RNA based vaccine	ARCT-021	2 doses Day 0 + Day 21 Intramuscular	Arcturus Therapeutics	Phase 2	Phase 1/2: NCT04480957
						Phase 2: NCT04668339 NCT04728347
23	Virus like particle	RBD SARS-CoV-2 HBsAg VLP vaccine	2 doses Day 0 + Day 28 Intramuscular	Serum Institute of India + Accelagen Pty + SpyBiotech	Phase 1/2	Phase 1/2: ACTRN12620000817943 ACTRN12620001308987
24	Inactivated virus	Inactivated SARS-CoV-2 vaccine (Vero cell)	2–3 doses Detailed schedule not specified Intramuscular	Beijing Minhai Biotechnology Co	Phase 2	Phase 1: NCT04758273
						Phase 2: NCT04756323
25	Viral vector (Non-replicating)	GRAd-COV2 (Replication defective Simian Adenovirus (GRAd) encoding S)	1 dose Day 0 Intramuscular	ReiThera + Leukocare + Univercells	Phase 2/3	Phase 1: NCT04528641
						Phase 2/3: NCT04791423
26	Viral vector (Non-replicating)	VXA-CoV2-1 Ad5 adjuvanted Oral Vaccine platform	2 doses Day 0 + Day 28 Intramuscular	Vaxart	Phase 1	Phase 1: NCT04563702
27	Viral vector (Non-replicating)	MVA-SARS-2-S	2 doses Day 0 + Day 28 Intramuscular	University of Munich (Ludwig-Maximilians)	Phase 1	Phase 1: NCT04569383
28	Protein subunit	SCB-2019 + AS03 or CpG 1018 adjuvant plus Alum adjuvant (Native like Trimeric subunit Spike Protein vaccine)	2 doses Day 0 + Day 21 Intramuscular	Clover Biopharmaceuticals Inc./GSK/Dynavax	Phase 2/3	Phase 1: NCT04405908
						Phase 2/3: NCT04672395
29	Protein subunit	COVAX-19^®^ Recombinant spike protein + adjuvant	2 doses Day 0 + Day 21 Intramuscular	Vaxine Pty Ltd.	Phase 1	Phase 1: NCT04453852
30	Protein subunit	MVC-COV1901 (S-2P protein + CpG 1018)	2 doses Day 0 + Day 28 Intramuscular	Medigen Vaccine Biologics + Dynavax + National Institute of Allergy and Infectious Diseases (NIAID)	Phase 2	Phase 1: NCT04487210
						Phase 2: NCT04695652
31	Protein subunit	FINLAY-FR1 anti-SARS-CoV-2 Vaccine (RBD + adjuvant)	2 doses Day 0 + Day 28 Intramuscular	Instituto Finlay de Vacunas	Phase 1/2	Phase 1: RPCEC00000338
						Phase 1/2: RPCEC00000332
32	Protein subunit	FINLAY-FR-2 anti-SARS-CoV-2 Vaccine (RBD chemically conjugated to tetanus toxoid plus adjuvant)	2 doses Day 0 + Day 28 Intramuscular	Instituto Finlay de Vacunas	Phase 3	Phase 1: RPCEC00000340
						Phase 2: RPCEC00000347
						Phase 3: RPCEC00000354
33	Protein subunit	EpiVacCorona (EpiVacCorona vaccine based on peptide antigens for the prevention of COVID-19)	2 doses Day 0 + Day 21 Intramuscular	Federal Budgetary Research Institution State Research Center of Virology and Biotechnology “Vector”	Phase 3	Phase 1/2: NCT04527575
						Phase 3: NCT04780035
34	Protein subunit	RBD (baculovirus production expressed in Sf9 cells) Recombinant SARS-CoV-2 vaccine (Sf9 Cell)	2 doses Day 0 + Day 21 Intramuscular	West China Hospital + Sichuan University	Phase 2	Phase 1: ChiCTR2000037518 NCT04530656
						Phase 2: ChiCTR2000039994 NCT04640402 NCT04718467
35	Protein subunit	IMP CoVac-1 (SARS-CoV-2 HLA-DR peptides)	1 dose Day 0 Subcutaneous	University Hospital Tuebingen	Phase 1	NCT04546841
36	Protein subunit	UB-612 (Multitope peptide based S1-RBD-protein based vaccine)	2 doses Day 0 + Day 28 Intramuscular	COVAXX + United Biomedical Inc	Phase 2/3	Phase 1: NCT04545749
						Phase 2: NCT04773067
						Phase 2/3: NCT04683224
37	Viral vector (Replicating)	DelNS1-2019-nCoV-RBD-OPT1 (Intranasal flu-based-RBD)	2 doses Day 0 + Day 28 Intranasal	University of Hong Kong, Xiamen University and Beijing Wantai Biological Pharmacy	Phase 2	Phase 1: ChiCTR2000037782 NCT04809389
						Phase 2: ChiCTR2000039715
38	RNA based vaccine	LNP-nCoVsaRNA	2 doses Day 0 + Day 28 Intranasal	Imperial College London	Phase 1	Phase 1: ISRCTN17072692
39	RNA based vaccine	SARS-CoV-2 mRNA vaccine (ARCoV)	2 doses Day 0 + Day 28 Intranasal	Academy of Military Science (AMS), Walvax Biotechnology and Suzhou Abogen Biosciences	Phase 2	Phase 1: ChiCTR2000034112 ChiCTR2000039212
						Phase 2: ChiCTR2100041855
40	Virus like particle	Coronavirus-Like Particle COVID-19 (CoVLP)	2 doses Day 0 + Day 21 Intranasal	Medicago Inc.	Phase 2/3	Phase 1: NCT04450004
						Phase 2: NCT04662697
						Phase 2/3: NCT04636697
41	Viral vector (Replicating) + APC	Covid-19/aAPC vaccine. The Covid-19/aAPC vaccine is prepared by applying lentivirus modification with immune modulatory genes and the viral minigenes to the artificial antigen presenting cells (aAPCs).	3 doses Day 0 + Day 14 + Day 28 Subcutaneous	Shenzhen Geno-Immune Medical Institute	Phase 1	Phase 1: NCT04299724
42	Viral vector (Non-replicating) + APC	LV-SMENP-DC vaccine. Dendritic cells are modified with lentivirus vectors expressing Covid-19 minigene SMENP and immune modulatory genes. CTLs are activated by LV-DC presenting Covid-19 specific antigens.	1 dose Day 0 Subcutaneous	Shenzhen Geno-Immune Medical Institute	Phase 1/2	Phase 1/2: NCT04276896
43	Protein subunit	AdimrSC-2f (Recombinant RBD +/− Aluminium)	No detail	Adimmune Corporation	Phase 1	Phase 1: NCT04522089
44	DNA based vaccine	Covigenix VAX-001—DNA vaccines + proteo-lipid vehicle (PLV) formulation	2 doses Day 0 + Day 14 Intramuscular	Entos Pharmaceuticals Inc.	Phase 1	NCT04591184
45	DNA based vaccine	CORVax—Spike (S) Protein Plasmid DNA Vaccine	2 doses Day 0 + Day 14 Intradermal	Providence Health & Services	Phase 1	Phase 1: NCT04627675
46	RNA based vaccine	ChulaCov19 mRNA vaccine	2 doses Day 0 + Day 21 Intramuscular	Chulalongkorn University	Phase 1	Phase 1: NCT04566276
47	DNA based vaccine	bacTRL-Spike oral DNA vaccine	1 dose Day 0 Oral	Symvivo Corporation	Phase 1	NCT04334980
48	Viral Vector (Non-replicating)	Human Adenovirus type 5: hAd5 S + N vaccine (S-fusion + N-ETSD) E2b-deleted Adeno	1–2 doses Day 0 + day 21 Subcutaneous or Oral	Immunity Bio.Inc	Phase 1	Phase 1: NCT04591717 NCT04710303
49	Viral vector (Non-replicating)	COH04S1 (MVA-SARS-2-S)—Modified vaccinia ankara (sMVA) platform + synthetic SARS-CoV-2	2 doses Day 0 + Day 28 Intramuscular	City of Hope Medical Center + National Cancer Institute	Phase 1	Phase 1: NCT04639466
50	Viral vector (Replicating)	rVSV-SARS-CoV-2-S Vaccine	1 dose Day 0 Intramuscular	Israel Institute for Biological Research	Phase 1/2	Phase 1/2: NCT04608305
51	Viral vector (Replicating) + APC	Dendritic cell vaccine AV-COVID-19. A vaccine consisting of autologous dendritic cells loaded with antigens from SARS-CoV-2, with or without GM-CSF	1 dose Day 0 Intramuscular	Aivita Biomedical, Inc. National Institute of Health Research and Development, Ministry of Health Republic of Indonesia	Phase 1/2	Phase 1: NCT04690387 NCT04685603
						Phase 1/2: NCT04386252
52	Live attenuated virus	COVI-VAC	1–2 doses Day 0 or Day 0 + 28 Intranasal	Codagenix/Serum Institute of India	Phase 1	Phase 1: NCT04619628
53	Protein subunit	CIGB-669 (RBD + AgnHB)	3 doses Day 0 + 14 + 28 or Day 0 + 28 + 56 Intranasal	Center for Genetic Engineering and Biotechnology (CIGB)	Phase 1/2	Phase 1/2: RPCEC00000345
54	Protein subunit	CIGB-66 (RBD + aluminium hydroxide)	3 doses Day 0 + 14 + 28 or Day 0 + 28 + 56 Intranasal	Center for Genetic Engineering and Biotechnology (CIGB)	Phase 3	Phase 1/2: RPCEC00000346
						Phase 3 RPCEC00000359
55	Inactivated Virus	VLA2001	2 doses Day 0 + Day 21 Intramuscular	Valneva, National Institute for Health Research, United Kingdom	Phase 1/2	Phase 1/2: NCT04671017
56	Protein subunit	BECOV2	2 doses Day 0 + Day 28 Intramuscular	Biological E. Limited	Phase 1/2	Phase 1/2: CTRI/2020/11/029032
57	Viral vector (Replicating)	AdCLD-CoV19 (adenovirus vector)	1 dose Day 0 Intramuscular	Cellid Co., Ltd.	Phase 1/2	Phase 1/2: NCT04666012
58	DNA based vaccine	GLS-5310	2 doses Day 0 + Day 56 or Day 0 + Day 84 Intradermal	GeneOne Life Science, Inc.	Phase 1/2	Phase 1/2: NCT04673149
59	Protein subunit	Recombinant Sars-CoV-2 Spike protein, Aluminum adjuvanted	2 doses Day 0 + 21 Intramuscular	Nanogen Pharmaceutical Biotechnology	Phase 1/2	Phase 1/2: NCT04683484
60	Protein subunit	Recombinant protein vaccine S-268019 (using Baculovirus expression vector system)	2 doses Day 0 + 21 Intramuscular	Shionogi	Phase 1/2	Phase 1/2: jRCT2051200092
61	Viral vector (Non-replicating)	AdCOVID, Adenovirus-based platform expresses the receptor-binding domain (RBD) of the Sars-Cov-2 spike protein	1 doses Day 0 Intranasal	Altimmune, Inc.	Phase 1	Phase 1: NCT04679909
62	Protein subunit	SARS-CoV-2-RBD-Fc fusion protein	Dosage and Schedule not specified Subcutaneous or intramuscular	University Medical Center Groningen + Akston Biosciences Inc.	Phase 1/2	Phase 1/2: NCT04681092
63	Inactivated Virus	ERUCOV-VAC, inactivated virus	2 doses Day 0 + 21 Intramuscular	Erciyes University	Phase 2	Phase 1: NCT04691947
						Phase 2: NCT04824391
64	Protein subunit	COVAC-1 and COVAC-2 sub-unit vaccine (spike protein) + SWE adjuvant	2 doses Day 0 + 28 Intramuscular	University of Saskatchewan	Phase 1/2	Phase 1/2: NCT04702178
65	Protein subunit	GBP510, a recombinant surface protein vaccine with adjuvant AS03 (aluminium hydroxide)	2 doses Day 0 + 28 Intramuscular	SK Biosciences Co. Ltd. and CEPI	Phase 1/2	Phase 1/2: NCT04742738 NCT04750343
66	Protein subunit	Razi Cov Pars, recombinant spike protein	3 doses Day 0 + 21 + 51 Intramuscular and intranasal	Razi Vaccine and Serum Research Institute	Phase 1	Phase 1: IRCT20201214049709N1
67	Inactivated Virus	COVID-19 inactivated vaccine	2 doses Day 0 + 14 Intramuscular	Shifa Pharmed Industrial Co	Phase 2/3	Phase 1: IRCT20201202049567N1 IRCT20201202049567N2
68	Protein subunit	MF59 adjuvanted SARS-CoV-2 Sclamp vaccine	2 doses Day 0 + 28 Intramuscular	The University of Queensland	Phase 1	Phase 1: NCT04495933
69	DNA based vaccine	COVIGEN	2 doses Day 0 + 28 Intramuscular or intradermal	University of Sydney, Bionet Co., Ltd. Technovalia	Phase 1	Phase 1: NCT04742842
70	DNA based vaccine	COVID-eVax, a candidate plasmid DNA vaccine of the Spike protein	No detailed dosage schedule Intramuscular	Takis + Rottapharm Biotech	Phase 1/2	Phase 1/2: NCT04788459 EUCTR2020-003734-20-IT
71	Viral vector (Non-replicating)	BBV154, Adenoviral vector COVID-19 vaccine	1 dose Day 0 Intramuscular	Bharat Biotech International Limited	Phase 1	Phase 1: NCT04751682
72	RNA based vaccine	PTX-COVID19-B, mRNA vaccine	2 doses Day 0 + 28 Intramuscular	Providence Therapeutics	Phase 1	Phase 1: NCT04765436
73	Viral vector (Replicating)	NDV-HXP-S, Newcastle disease virus (NDV) vector expressing the spike protein of SARS-CoV-2, with or without the adjuvant CpG 1018	2 doses Day 0 + 28 Intramuscular	Mahidol University; The Government Pharmaceutical Organization (GPO); Icahn School of Medicine at Mount Sinai	Phase 1/2	Phase 1/2: NCT04764422
74	RNA based vaccine	CoV2 SAM (LNP) vaccine. A self-amplifying mRNA (SAM) lipid nanoparticle (LNP) platform + Spike antigen	2 doses Day 0 + 28 Intramuscular	GlaxoSmithKline	Phase 1	Phase 1: NCT04758962
75	Virus like particle	VBI-2902a. An enveloped virus-like particle (eVLP) of SARS-CoV-2 spike (S) glycoprotein and aluminum phosphate adjuvant.	2 doses Day 0 + 28 Intramuscular	VBI Vaccines Inc.	Phase 1/2	NCT04773665
76	Protein subunit	SK SARS-CoV-2 recombinant surface antigen protein subunit (NBP2001) + adjuvanted with alum.	2 doses Day 0 + 28 Intramuscular	SK Bioscience Co., Ltd.	Phase 1	Phase 1: NCT04760743
77	Viral vector (Non-replicating)	Chimpanzee Adenovirus serotype 68 (ChAd) and self-amplifying mRNA (SAM) vectors expressing spike alone, or spike plus additional SARS-CoV-2 T cell epitopes.	2–3 doses Day 0 +14 + 28 or Day 0 + 28 + 56 or Day 0 + 112 Intramuscular	Gritstone Oncology	Phase 1	Phase 1: NCT04776317
78	RNA based vaccine	mRNA-1273.351. A lipid nanoparticle (LNP)-encapsulated mRNA-based vaccine that encodes for a full-length, prefusion stabilized S protein of the SARS-CoV-2 B.1.351 variant.	3 doses Day 0 + 28 + 56 Intramuscular	Moderna + National Institute of Allergy and Infectious Diseases (NIAID)	Phase 1	Phase 1: NCT04785144
79	Protein subunit	SpFN (spike ferritin nanoparticle) uses spike proteins with a liposomal formulation QS21 (ALFQ) adjuvant.	3 doses Day 0 + 28 + 120 Intramuscular	Walter Reed Army Institute of Research (WRAIR)	Phase 1	Phase 1: NCT04784767
80	Protein subunit	EuCorVac-19; A spike protein using the recombinant protein technology and with an adjuvant.	2 doses Day 0 + 21 Intramuscular	POP Biotechnologies and EuBiologics Co.,Ltd	Phase 1/2	Phase 1/2: NCT04783311
81	Inactivated virus	Inactivated SARS-CoV-2 vaccine FAKHRAVAC (MIVAC)	2 doses Day 0 + 21 Intramuscular	Organization of Defensive Innovation and Research	Phase 1	Phase 1: IRCT20210206050259N1
82	Live attenuated virus	MV-014-212, a live attenuated vaccine that expresses the spike (S) protein of SARS-CoV-2	2 doses Day 0 + 35 Intranasal	Meissa Vaccines, Inc.	Phase 1	Phase 1: NCT04798001
83	RNA based vaccine	MRT5500, an mRNA vaccine candidate	2 doses Day 0 + 21 Intramuscular	Sanofi Pasteur and Translate Bio	Phase 1/2	Phase 1/2: NCT04798027
84	Virus like particle	SARS-CoV-2 VLP Vaccine	1 doses Day 0 Subcutaneous	The Scientific and Technological Research Council of Turkey	Phase 1	Phase 1: NCT04818281
85	Protein subunit	ReCOV: Recombinant two-component spike and RBD protein COVID-19 vaccine (CHO cell).	2 doses Day 0 + 21 Intramuscular	Jiangsu Rec-Biotechnology	Phase 1	Phase 1: NCT04818801
86	RNA based vaccine	DS-5670a, mRNA vaccine	2 doses Day 0 + 21 Intramuscular	Daiichi Sankyo Co., Ltd.	Phase 1/2	Phase 1/2: NCT04821674

## Data Availability

The data generated during and/or analysed during the current study are available on electronic databases (PubMed, Embase, Medline, Google Scholar, Cochrane). All data generated or analysed during this study are included in this published article.
